# LLMs uncover 6M1@C60’s therapeutic targets: inhibiting atherosclerotic progression via NRF2/HO-1/GPX4 axis-mediated ferroptosis suppression

**DOI:** 10.1186/s12951-026-04474-3

**Published:** 2026-06-02

**Authors:** Wenhao Liu, Guofeng Zhou, Xin Gao, Bo Li, Yingjie Zhao

**Affiliations:** 1 Department of Cardiology, Zibo Central Hospital, No. 10, South Shanghai Road, Zibo, 255000 Shandong Province China; 2 Department of Dermatology, Zibo Central Hospital, Zibo, 255000 China; 3 College of Polymer Science and Engineering, Qingdao University of Science and Technology, No. 99, Songling Road, Qingdao, 266000 Shandong Province China

**Keywords:** Fullerene, Ferroptosis, Large Language Model, Atherosclerosis, Nuclear Factor Erythroid 2-Related Factor 2, Biomaterials

## Abstract

Biomaterials have underpinned tissue engineering, drug delivery, and disease treatment for over 80 years, yet their clinical translation is hampered by unclear molecular mechanisms. Fullerene derivatives like 6M1@C60 exhibit antioxidant/anti-inflammatory potential for atherosclerosis but lack defined therapeutic targets. We integrated multi-databases (GeneCards, TTD, OMIM, CTD) and used ChatGPT-4 with chain-of-thought reasoning to predict 6M1@C60 targets; 11 candidates were identified, and NRF2 was experimentally validated. In ApoE^⁻/⁻^ mice, high-dose 6M1@C60 reduced aortic plaque area, lowered ferroptosis markers (4-HNE, MDA, Fe²⁺), and improved oxidative stress (reduced TG, TC, LDL, ox-LDL). In vitro, it suppressed Erastin-induced RAW264.7 macrophage ferroptosis (decreased ROS/LPO, increased GSH/GSSG)—effects reversed by the NRF2 inhibitor ML385 and rescued by GPX4 overexpression. Linking LLM-driven prediction to mechanistic validation, we show 6M1@C60 attenuates atherosclerosis via NRF2/HO-1/GPX4-mediated ferroptosis regulation. These findings provide a theoretical basis for fullerene clinical application and highlight LLMs’ utility in biomaterial target discovery.

**Graphical Abstract:**

**Figure:**
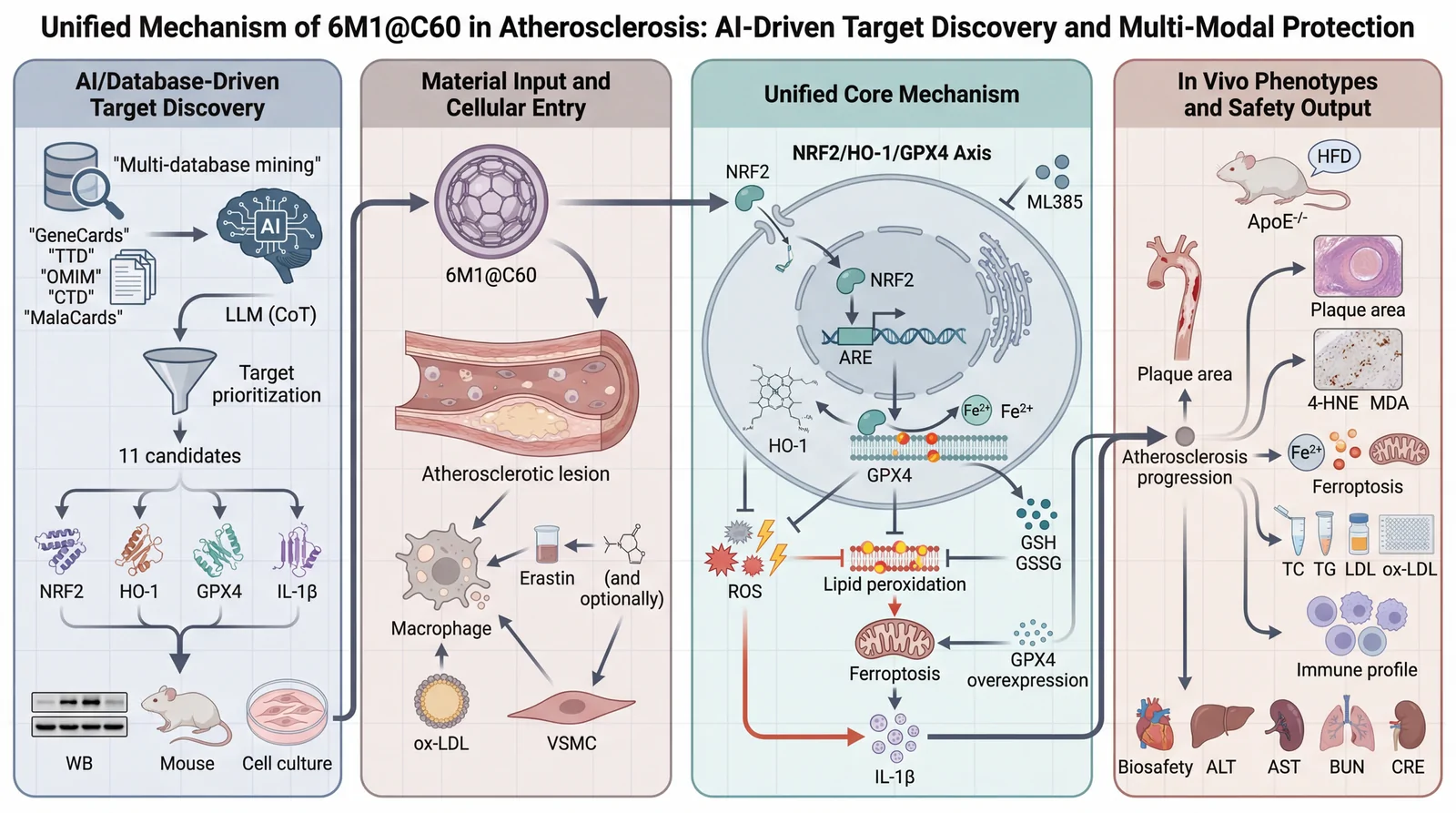

**Supplementary Information:**

The online version contains supplementary material available at 10.1186/s12951-026-04474-3.

## Introduction

Atherosclerosis is widely recognized as a lipid-driven, low-grade chronic inflammatory disease characterized by a highly complex pathogenesis involving the interplay of oxidative stress, inflammatory responses, and immune activation [1–3]. Pathological studies have shown that atherosclerosis initiates with the deposition of oxidized lipids within the arterial intima. These lipids are recognized and engulfed by monocytes, which differentiate into macrophages and subsequently form foam cells, establishing a plaque microenvironment rich in reactive oxygen species (ROS) and pro-inflammatory factors [4–6]. This imbalance between oxidative stress and inflammation persistently activates local immune cells, exacerbating plaque formation and arterial narrowing [7, 8]. As a result, considerable research efforts have been devoted to developing novel therapeutic strategies to modulate ROS accumulation and inflammatory responses to slow or even reverse atherosclerotic progression—an area of immense clinical potential and medical relevance.

Biomaterials have emerged as essential enablers of precision medicine in the evolving landscape of translational medicine due to their excellent biocompatibility and multi-functionality. In recent years, they have achieved significant progress in tissue engineering, drug delivery, and regenerative therapies, offering new paradigms for treating various diseases [9–11]. However, traditional research has predominantly focused on these materials’ physicochemical properties and biosafety, often overlooking their intricate interactions with cellular signaling pathways and disease mechanisms. This knowledge gap has become a major barrier to their clinical translation [12]. With advances in nanotechnology and molecular biology, novel nanomaterials exhibit enhanced targeting capabilities and tunable functionalities, presenting new opportunities to overcome current limitations and drive mechanistic research and translational applications of biomaterials in complex disease contexts.

Fullerene, a representative class of carbon-based nanomaterials, has garnered considerable attention in the biomedical field due to its unique spherical conjugated structure and exceptional free radical scavenging capacity [13–15]. In particular, fullerene and its easily modifiable derivatives exhibit favorable biocompatibility and outstanding antioxidant activity and have been increasingly studied in recent years for their therapeutic potential in diseases such as type 2 diabetes, hepatic steatosis, ulcerative colitis, and atherosclerosis [16–20]. However, their precise molecular targets and mechanisms of action in vivo remain unclear, limiting clinical translation. Functionalized fullerene derivatives such as 6M1@C60 have recently emerged, offering improved water solubility and targeting ability, thereby providing new opportunities for mechanistic research and therapeutic development.

Ferroptosis is an iron-dependent form of regulated cell death driven by lipid peroxidation (LPO), which has gained significant attention in cardiovascular research in recent years [21–23]. In the context of atherosclerosis, ferroptosis is implicated in endothelial dysfunction, the formation of an inflammatory microenvironment, and plaque instability. In particular, disrupted iron metabolism and the dysfunction of antioxidant systems such as glutathione peroxidase 4 (GPX4) in macrophages and foam cells are known to promote plaque progression [24–26]. The nuclear factor erythroid 2-related factor 2/heme oxygenase 1(NRF2/HO-1)/GPX4 signaling axis serves as a central regulatory pathway in ferroptosis by maintaining cellular redox homeostasis and glutathione (GSH) metabolism and has emerged as a critical therapeutic target [27, 28]. Therefore, targeting this pathway represents a promising strategy for preventing and treating atherosclerosis.

With the continuous advancement of target identification technologies, the application of artificial intelligence (AI)—particularly large language models (LLMs)—has opened new avenues for investigating complex disease mechanisms and predicting the therapeutic targets of novel biomaterials [29, 30]. Compared to traditional approaches, LLMs such as ChatGPT possess multimodal understanding, database integration, and reasoning capabilities, enabling efficient identification of potential targets and construction of disease-drug association networks [31, 32]. Building on this technology, the present study integrates data from GeneCards, the Therapeutic Target Database (TTD), MalaCards, Online Mendelian Inheritance in Man (OMIM), and the Comparative Toxicogenomics Database (CTD) to systematically construct a molecular association framework between fullerene derivatives and atherosclerosis, followed by experimental validation of the underlying mechanisms.

In summary, this study focuses on the functionalized fullerene derivative 6M1@C60 and employs LLM-based target prediction to systematically investigate its potential mechanism of action in atherosclerosis, particularly emphasizing its role in modulating ferroptosis. By incorporating ChatGPT-4.0 and chain-of-thought (CoT) reasoning strategies, we integrate multi-omics database information to construct a molecular network linking material, target, and disease. Based on this framework, in vitro and in vivo experiments are designed to verify key signaling pathways and explore the underlying mechanisms. Theoretically, this work enhances our mechanistic understanding of fullerene in cardiovascular disease treatment; methodologically, it establishes a novel paradigm that integrates AI with biomaterials research, offering a data-driven pathway for the intelligent design and precision therapy of new materials with strong clinical translational potential.

## Materials and methods

### Reagents

Oxidized low-density lipoprotein (ox-LDL) was purchased from Yiyuan Biotechnologies (Guangzhou, China). Erastin and ML385 were obtained from AdooQ BioScience (USA). The primary antibodies were sourced as follows: TXN (R27465), GCLC (R24420), NRF2 (R380773), PARK7 (R22793), P53 (R25247), IL-6 (347023), IL-1β (511369), MAPK3 (343830), HO-1 (R24541), and RelA (380172) were purchased from ZEN-BIOSCIENCE (Chengdu, China). The GPX4 antibody (ab125066) was purchased from Abcam (Cambridge, UK).

## Data collection and enrichment analysis

Protein targets and pharmacological mechanisms related to fullerene and its analogs were collected and categorized. All targets were standardized to human proteins and submitted to the Database for Annotation, Visualization and Integrated Discovery (DAVID, https://david.ncifcrf.gov/) for enrichment analysis. Both Gene Ontology (GO) and Kyoto Encyclopedia of Genes and Genomes (KEGG) analyses were conducted using a significance threshold of *p* < 0.05.

### Animal experiments

A total of 35 healthy male apolipoprotein E knockout (ApoE^⁻/⁻^) mice (8 weeks old; C57BL/6 background) were purchased from Ji’nan Pengyue Laboratory Animal Breeding Co., Ltd. and housed under specific pathogen-free conditions at 25 ± 2 °C and 50–60% humidity. Lentiviral vectors (oe-NC or oe-GPX4) were administered via tail-vein injection, followed by a 1-week acclimation period. Mice were then randomized into seven groups (*n* = 5 per group) using a computer-generated sequence prepared and securely retained by an investigator not involved in subsequent interventions or outcome assessments. All procedures, including drug (or vehicle) administration, behavioral testing, tissue collection and primary measurements, were performed by personnel blinded to group allocation; samples were coded and unblinding was conducted only after completion of core data collection and prior to final statistical analysis.

The dose of 6M1@C60 had been evaluated in prior toxicity testing (Table S1). Group allocation was as follows: (1) the normal group (Normal), in which mice were fed a standard diet as a control; (2) the model group (HFD), in which mice were fed a high-fat diet (HFD) and intraperitoneally injected with 200 µL of saline twice per week for 12 weeks as a placebo control; (3) the low-dose treatment group (HFD + 6M1@C60-L), in which mice were fed a HFD and administered 200 µL of low-dose 6M1@C60 via oral gavage twice per week for 12 weeks (20 mg/L); (4) the high-dose treatment group (HFD + 6M1@C60-H), in which mice were fed a HFD and administered 200 µL of high-dose 6M1@C60 (40 mg/L) via oral gavage twice per week for 12 weeks; (5) and the HFD + 6M1@C60-H + ML385 group, in which mice were fed a HFD and administered 200 µL of low-dose 6M1@C60 (20 mg/L) via oral gavage twice per week for 12 weeks. ML385 (30 mg/kg) was dissolved in corn oil containing 5% dimethyl sulfoxide (DMSO) and delivered via intraperitoneal injection; (6) the HFD + 6M1@C60-H + ML385 + oe-NC group, in which mice were fed a HFD, treated with low-dose 6M1@C60 (20 mg/L), and injected intraperitoneally with ML385 prepared in 5% DMSO corn oil after oe-NC transduction; (7) the HFD + 6M1@C60-H + ML385 + oe-GPX4 group, in which mice were treated in the same manner, but received lentiviral oe-GPX4 transduction instead [33]. At the end of the experiment, mice were euthanized, and perfusion was performed through the left ventricle using 4% paraformaldehyde. The aorta and major organs were collected and preserved according to the specific needs of downstream experiments.

The entire aorta was carefully cleaned to remove surrounding adipose tissue, longitudinally opened, and fixed. Oil Red O staining (Solarbio, China) was performed to quantify the plaque area. Digital images were captured using Image-Pro Plus 6.0 software and analyzed quantitatively.

The aortic root was embedded in an optimal cutting temperature (OCT) compound and frozen for cryosectioning. Serial frozen sections with a thickness of 5 μm were prepared and stored at -20 °C for subsequent analysis. According to the manufacturer’s instructions, hematoxylin and eosin (H&E) staining (Solarbio, Beijing, China), Masson’s trichrome staining (LEAGENE, Beijing, China), and Oil Red O staining (Solarbio, Beijing, China) were performed. Quantitative image analysis was conducted using Image-Pro Plus 6.0 software.

### Measurement of clood lipid parameters (Total Cholesterol (TC), Triglycerides (TG), and ox-LDL)

Mice were anaesthetized with isoflurane delivered via inhalation. After a 12-h fast, blood was collected by cardiac puncture into anticoagulant tubes and centrifuged at 4℃ to obtain serum. Serum concentrations of total cholesterol (TC; A111-1-1), triglycerides (TG; A110-1-1) and oxidized low-density lipoprotein (ox-LDL; A113-1-1) were quantified using the corresponding assay kits (Nanjing Jiancheng Bioengineering Institute, China) according to the manufacturer’s instructions.

### Immunohistochemistry (IHC)

Aortas were collected, fixed in 4% paraformaldehyde, embedded in paraffin, and sectioned. Sections were deparaffinized in xylene, rehydrated through a graded ethanol series, and incubated in 3% H_2_O_2_ in methanol for 15 min to block endogenous peroxidase activity. After blocking with BSA for 30 min, the sections were incubated overnight at 4 °C in a humidified chamber with the 4-hydroxynonenal (4-HNE) primary antibody (MA5-27570, 1:1000, Thermo Fisher). DAB staining and hematoxylin counterstaining were performed, and the sections were sealed with a neutral mounting medium and visualized under a light microscope. Immunostaining was evaluated using Image-Pro Plus 6.0 software by selecting a consistent brown color threshold to define positive staining. Integrated optical density (IOD) and stained area were quantified for each image.

### Cell culture and lentiviral infection

RAW264.7 and vascular smooth muscle cell (VSMC) lines were obtained from iCell Bioscience Inc. (Shanghai, China) and cultured in Dulbecco’s modified Eagle’s medium (DMEM; Gibco, Beijing, China) supplemented with 10% fetal bovine serum (Gibco, Beijing, China) and 1% penicillin–streptomycin (Biosharp, Beijing, China). Lentiviral particles carrying oe-NC, oe-GPX4, si-NRF2, or si-GPX4 were packaged in HEK-293T cells (CRL-11268; ATCC) (Table S2). The pLenti6/V5-DEST™ Gateway™ vector (V49610; Thermo Fisher) was used for lentiviral overexpression. Viral supernatants were collected 48 h after transfection, and virus concentration was performed by Genechem (Shanghai, China). RAW264.7 cells at ~ 50% confluence were infected with lentivirus at an MOI of 5; 48 h post-infection, cells were selected with puromycin (10 µg/mL; HY-K1057; MCE) and maintained for at least 1 week to establish stable cell lines [34]. RAW264.7 and VSMCs were incubated with erastin (5 µM) for 12 h and subsequently treated with 6M1@C60 at low (30 nM) or high (60 nM) concentrations.

For the luciferase reporter assay [35], the GPX4-ARE plasmid was kindly provided by Being-Sun Wung (National Chiayi University, Chiayi, Taiwan). ARE transcriptional activity was measured using the Dual-Luciferase Reporter (DLR) Assay System (Promega, CA, USA). Cells were transfected with the GPX4-ARE luciferase reporter construct using PureFection transfection reagent. After 4 h, the medium was replaced and cells were treated with the indicated compounds for 24 h. Luciferase activity was quantified using a microplate luminometer (Turner Biosystems, Sunnyvale, CA, USA).

### Lipid detection in cells

Lipid accumulation in RAW264.7 cells was assessed using an Oil Red O staining kit (Solarbio, Beijing, China). Before staining, cells were treated with 50 µg/mL ox-LDL for 24 h.

### Detection of intracellular ROS

Intracellular ROS levels were detected using the DCFH-DA probe (Beyotime, Shanghai, China). DCFH-DA was diluted in serum-free medium to a final concentration of 10 µM, and all procedures were carried out under light-protected conditions. Overconcentration was avoided to prevent excessive background fluorescence. Cells were washed three times with 1× phosphate-buffered saline (PBS) to minimize serum interference and incubated for 40 min in a cell incubator. ROS fluorescence intensity in each well was then observed under a fluorescence inverted microscope (Invitrogen, USA), with consistent exposure time applied across all wells and protection from light maintained throughout.

### Transmission electron microscopy (TEM)

After drug treatment, RAW264.7 cells were washed three times with PBS buffer to remove the residual culture medium. The cells were then fixed in a TEM fixation solution for 25 min. After fixation, the samples were dehydrated, infiltrated with wax, and embedded in paraffin. After staining, the samples were examined under TEM.

### Cell apoptosis assay

RAW264.7 cells were seeded into 6-well plates at a density of 1 × 10⁶ cells per well and incubated overnight. After 24 h of drug treatment, the cells were harvested, washed twice with PBS, and resuspended in 1 mL of Annexin V binding buffer. The cell suspension was then incubated with 10 µL of Annexin V-FITC/propidium iodide (PI) dye for 20 min at room temperature. Apoptosis was assessed using flow cytometry.

### Flow cytometry

Single-cell suspensions from blood, bone marrow and spleen were generated by mincing tissues and digesting in collagenase (2 mg/mL; 17018029, Sigma) at 37 °C for 1 h, followed by filtration through a 70-µm strainer. After red blood cell lysis (C3702, Beyotime; 5 min), cells were fixed (0.5 mL; 420801, BioLegend) for 20 min at room temperature, washed in 1× PBS containing 2% BSA, and permeabilized using intracellular staining permeabilization buffer (421002, BioLegend). Cells were stained with anti-F4/80–PE (1:100, T45-2342) and antibodies against CD8 (ab217344, Abcam), CD68 (ab283654, Abcam), CD11b (ab8878, Abcam), CD4 (ab133616, Abcam) and FOXP3 (ab215206, Abcam) for 30 min at room temperature, washed, and analyzed by flow cytometry. Intact leukocytes were gated by FSC/SSC, and population frequencies were quantified from fluorescence intensity distributions [36, 37].

### Cell viability assay

Cell proliferation was evaluated using the Cell Counting Kit-8 (CCK-8) assay. RAW264.7 cells were seeded into 96-well plates (Corning Corporation, Corning, New York) at a density of 3,000 cells per well. After the indicated treatments, 10 µL of CCK-8 solution was added to each well and incubated for 1 h. Absorbance was measured at 450 nm using a microplate reader.

### LPO assay in cells

RAW264.7 cells were seeded into 6-well plates at a density of 1 × 10⁶ cells per well and incubated overnight. After 24 h of treatment, cells were washed once with serum-free DMEM, harvested, and exposed to 2 µM C11 BODIPY 581/591 (Invitrogen) to assess LPO. After two washes with PBS, the cells were collected and analyzed using flow cytometry. The fluorescent signal of C11 BODIPY 581/591 was detected in the FL1 channel.

### Determination of GSH/Glutathione Disulfide (GSSG), Fe²⁺, and Malondialdehyde (MDA) Levels

All assays were performed according to the instructions provided with each commercial kit. MDA levels were measured using an MDA assay kit (S0131, Beyotime, Shanghai, China) based on the reaction between MDA and thiobarbituric acid (TBA). Reduced GSH levels were determined using a GSH assay kit (S0053, Beyotime), in which GSH reacts with DTNB to form 2-nitro-5-thiobenzoic acid and GSSG. The concentration of Fe²⁺ was assessed using an iron assay kit (ab83366, Abcam), which detects Fe²⁺ through the formation of a stable colored complex with Ferene S.

### Biochemical assays and in vivo biodistribution

All measurements were performed according to the manufacturers’ protocols. NADPH levels were quantified using an NADP⁺/NADPH Assay Kit (S0179; Beyotime, Shanghai, China); SOD activity using an SOD Activity Assay Kit (SOD; Beyotime, Shanghai, China); CAT activity using a Catalase (CAT) Assay Kit (S0051; Beyotime, Shanghai, China); and GSH-PX activity using a Glutathione Peroxidase (GSH-PX) Assay Kit (S0056; Beyotime, Shanghai, China). Serum samples were collected before euthanasia, and alanine aminotransferase (ALT) and aspartate aminotransferase (AST) were measured to evaluate hepatocellular injury, while blood urea nitrogen (BUN) and creatinine (CRE) were measured to evaluate renal injury, using commercial kits (Nanjing Jiancheng Bioengineering Institute, China) according to the manufacturer’s instructions. For biodistribution analysis, mice received a tail-vein injection of 150 µL DiD-labelled 6M1@C60 (3 mg/mL); at 48 h post-injection, five mice were euthanized and the liver, kidney, spleen, lung and heart were harvested, weighed and homogenized in 1 mL PBS, and fluorescence intensity was quantified using an Infinite M200 microplate reader.

### Prompting strategy

A two-round prompting workflow was applied to GPT-4.0 for candidate target prioritization. In round 1, the model was queried with the core scientific question and each output was manually assessed; vague answers or statements lacking verifiable support were excluded, whereas unambiguous outputs were advanced to round 2. In round 2, additional contextual constraints were provided as priors and the model was instructed to return structured justification (key biological links, retrievable evidence cues, and testable predictions) to improve interpretability and consistency. Key prompt outputs were cross-checked using an updated GPT-5.0 model, yielding concordant major candidates and mechanistic inferences. Prioritization criteria included: (1) functional relevance to ferroptosis and atherosclerosis; (2) supporting evidence in the literature or databases; and (3) consistency across repeated queries. The model reported confidence scores on a 0–100 scale; proteins with scores ≥ 80 that recurred across queries were defined as high-confidence candidates. Eleven high-confidence targets were subsequently evaluated by western blot, and four exhibited significant expression changes consistent with the model predictions, supporting the robustness and reproducibility of the approach. A representative two-round prompting example is provided below.

### Round 1

Prompt 1 (provided by researchers):

Can you explain the role of the protein “GPX4” in ferroptosis during atherosclerosis?

Response 1 (provided by GPT-4.0):

GPX4 is critical in preventing ferroptosis, a regulated cell death driven by iron-dependent LPO. In atherosclerosis, GPX4 is particularly important as it protects cells—especially vascular endothelial and smooth muscle cells—from oxidative damage that contributes to disease progression.

Functions of GPX4 in Ferroptosis:

Antioxidant defense: GPX4 reduces lipid hydroperoxides to their corresponding alcohols and prevents the formation of lipid radicals, which are key drivers of ferroptosis. Through this mechanism, GPX4 maintains the integrity of cellular membranes and inhibits LPO-induced cell death.

GSH dependence: GPX4 uses GSH as a cofactor to detoxify lipid hydroperoxides. GSH availability is crucial for GPX4 activity; depletion of GSH impairs GPX4 function and promotes ferroptosis.

LPO prevention: By directly reducing phospholipid hydroperoxides, GPX4 prevents the accumulation of toxic lipid peroxides in cell membranes, which would otherwise trigger ferroptotic cell death.

Role of GPX4 in Atherosclerosis:

Protection of vascular cells: In atherosclerosis, oxidative stress plays a central role in endothelial dysfunction, smooth muscle cell death, and foam cell formation. GPX4 protects these vascular cells from ferroptosis, thereby attenuating the progression of atherosclerotic lesions.

Prevention of plaque formation: By inhibiting ferroptosis in vascular cells, GPX4 prevents their necrosis, which is a critical event in the formation of necrotic cores within atherosclerotic plaques. These necrotic cores contribute to plaque instability, increasing the risk of rupture and subsequent cardiovascular events.

Regulation of inflammation: Ferroptosis can trigger inflammation by releasing damage-associated molecular patterns (DAMPs). GPX4, by suppressing ferroptosis, also reduces the associated inflammatory response, which is a key component in the progression of atherosclerosis.

In summary, GPX4 functions as a protective enzyme in atherosclerosis by reducing LPO, safeguarding vascular cells from oxidative damage, and contributing to plaque stability.

### Round 2

Prompt 2 (provided by researchers):

IL6 (interleukin-6), TNF (tumor necrosis factor), CASP3 (caspase-3), AKT1 (RAC-α serine/threonine-protein kinase), AKT2 (RAC-β serine/threonine-protein kinase), AKT3 (RAC-γ serine/threonine-protein kinase), NFE2L2 (nuclear factor erythroid 2-related factor 2), HMOX1 (heme oxygenase 1), CYCS (cytochrome c), BECN1 (Beclin-1), TP53 (tumor protein p53), SOD2 (superoxide dismutase [Mn], mitochondrial), GPX4 (phospholipid hydroperoxide glutathione peroxidase), GSTP1 (glutathione S-transferase P), GCLC (glutamate-cysteine ligase catalytic subunit), COX2 (cytochrome c oxidase subunit 2), BCL2 (B-cell lymphoma 2), MAP1LC3B (microtubule-associated protein 1 A/1B-light chain 3B), MAPK1 (mitogen-activated protein kinase 1), MAPK3 (mitogen-activated protein kinase 3), MAPK11 (mitogen-activated protein kinase 11), MAPK12 (mitogen-activated protein kinase 12), MAPK14 (mitogen-activated protein kinase 14), TNNT2 (troponin T type 2), ACTN2 (α-actinin-2), GJA1 (gap junction alpha-1 protein), BRCA1 (breast cancer type 1 susceptibility protein), BAX (Bcl-2-associated X protein), RELA (transcription factor p65), IL1A (interleukin-1 alpha), VEGFA (vascular endothelial growth factor A), VEGFR2 (vascular endothelial growth factor receptor 2), HRH1 (histamine H1 receptor), HTR4 (5-hydroxytryptamine receptor 4), OCLN (occludin), CLDN3 (claudin-3), CLDN5 (claudin-5), VCAM1 (vascular cell adhesion molecule 1), ICAM1 (intercellular adhesion molecule 1), IFNG (interferon gamma), IL1B (interleukin-1 beta), IL12A (interleukin-12 subunit alpha), IL12B (interleukin-12 subunit beta), GRM1 (metabotropic glutamate receptor 1), ADORA1 (adenosine A1 receptor), ADORA2A (adenosine A2a receptor), MMP1 (matrix metallopeptidase 1), MMP3 (stromelysin-1), MMP13 (collagenase 3), TRPM2 (transient receptor potential cation channel subfamily M member 2), GRIA1 (glutamate ionotropic receptor AMPA type subunit 1), CAMK2A (calcium/calmodulin-dependent protein kinase II subunit alpha), CREB1 (cAMP response element-binding protein 1), IL4 (interleukin-4), IL5 (interleukin-5), FOXP3 (forkhead box protein P3), FLG (filaggrin), HMGB1 (high mobility group box 1), RAGE (receptor for advanced glycation end products), RAC1 (Ras-related C3 botulinum toxin substrate 1), MMP2 (72 kDa type IV collagenase), TIMP2 (tissue inhibitor of metalloproteinases 2), MMP9 (matrix metalloproteinase 9), TIMP1 (tissue inhibitor of metalloproteinases 1), TIMP4 (tissue inhibitor of metalloproteinases 4), IKBKB (inhibitor of nuclear factor kappa-B kinase subunit beta), NFKBIA (nuclear factor of kappa light polypeptide gene enhancer in B-cells inhibitor, alpha), and ZO1 (tight junction protein ZO-1) have been identified as targets of C60 and its derivatives. 6M1@C60 is a classical derivative of C60 that significantly enhances its water solubility without altering the original structure or biomedical activity of C60. The question is: Does GPX4 participate in the pharmacological mechanism of 6M1@C60?

Response 2 (provided by GPT-4.0):

The pharmacological mechanism of 6M1@C60, a water-soluble derivative of C60, involves multiple molecular targets, including GPX4. GPX4 is a critical enzyme that prevents ferroptosis by reducing lipid hydroperoxides to their corresponding alcohols, thereby protecting cells from oxidative stress-induced cell death.

Given that 6M1@C60 has been shown to interact with numerous proteins associated with oxidative stress, apoptosis, and inflammation, GPX4 may participate in its pharmacological mechanism through the following pathways:

Potential roles of GPX4 in the mechanism of 6M1@C60:

Protection against oxidative stress: 6M1@C60 possesses antioxidant properties, and GPX4 is key in neutralizing lipid peroxides. The interaction between 6M1@C60 and GPX4 may support or enhance GPX4 activity, thereby increasing its capacity to mitigate oxidative stress and prevent ferroptosis.

Regulation of ferroptosis: Since GPX4 is a central player in the process, 6M1@C60 may exert its protective effects through interaction with or upregulation of GPX4. This mechanism may be particularly relevant in pathological conditions such as atherosclerosis, where oxidative stress and ferroptosis contribute to disease progression.

Synergistic antioxidant effects: 6M1@C60’s antioxidant activity may act synergistically with GPX4, enhancing cellular defense against LPO and oxidative damage. This synergy could underlie the observed anti-apoptotic and anti-inflammatory effects of 6M1@C60.

In summary, GPX4 is likely an important component of the pharmacological mechanism 6M1@C60, especially in mediating its antioxidant and anti-ferroptotic effects. The interaction between 6M1@C60 and GPX4 may be a key factor in the compound’s protective actions across various biological contexts.

### Round 3

Prompt 3 (provided by researchers):

GPX4:

GPX4 prevents ferroptosis, a regulated cell death driven by iron-dependent LPO. In atherosclerosis, GPX4 is particularly important as it protects cells—especially vascular endothelial and smooth muscle cells—from oxidative damage that contributes to disease progression.

Functions of GPX4 in Ferroptosis:

Antioxidant defense: GPX4 reduces lipid hydroperoxides to their corresponding alcohols and prevents the formation of lipid radicals, which are key drivers of ferroptosis. In doing so, GPX4 preserves the integrity of cellular membranes and inhibits LPO-induced cell death.

GSH dependence: GPX4 utilizes GSH as a cofactor to detoxify lipid hydroperoxides. GSH availability is essential for GPX4 activity; depletion of GSH impairs GPX4 function and promotes ferroptosis.

Prevention of LPO: By directly reducing phospholipid hydroperoxides, GPX4 prevents the accumulation of toxic lipid peroxides in the cell membrane, which would otherwise trigger ferroptotic cell death.

Role of GPX4 in Atherosclerosis:

Protection of vascular cells: In atherosclerosis, oxidative stress plays a central role in endothelial dysfunction, smooth muscle cell death, and foam cell formation. GPX4 mitigates the progression of atherosclerotic lesions by protecting these vascular cells from ferroptosis.

Prevention of plaque formation: By inhibiting ferroptosis in vascular cells, GPX4 prevents their necrosis—a key event in the development of necrotic cores within atherosclerotic plaques. These necrotic cores contribute to plaque instability, possibly leading to plaque rupture and cardiovascular events.

Regulation of inflammation: Ferroptosis can trigger inflammation through the release of DAMPs. GPX4, by suppressing ferroptosis, also reduces inflammation, a critical component of atherosclerosis progression.

In summary, through its ability to reduce lipid peroxides, GPX4 functions as a key protective enzyme in atherosclerosis by shielding vascular cells from oxidative damage and contributing to plaque stability.

GPX4 and 6M1@C60: The pharmacological mechanism of 6M1@C60, a water-soluble derivative of C60, involves multiple molecular targets, including GPX4. As a key enzyme, GPX4 prevents ferroptosis by reducing lipid hydroperoxides to their corresponding alcohols, thereby protecting cells from oxidative stress-induced death.

Given that 6M1@C60 has been shown to target multiple proteins associated with oxidative stress, apoptosis, and inflammation, GPX4 may be involved in its pharmacological mechanism through the following pathways:

Potential Roles of GPX4 in the Mechanism of 6M1@C60:

Protection against oxidative stress: 6M1@C60 possesses antioxidant properties, while GPX4 is critical in neutralizing lipid peroxides. The interaction between 6M1@C60 and GPX4 may support or enhance GPX4 activity, thereby improving its ability to reduce oxidative stress and prevent ferroptosis.

Regulation of ferroptosis: As a central ferroptosis regulator, GPX4 may be influenced by 6M1@C60 either through direct interaction or upregulation. This mechanism may help prevent cell death under pathological conditions such as atherosclerosis, where oxidative stress and ferroptosis contribute to disease progression.

Synergistic antioxidant effects: 6M1@C60’s antioxidant activity may act synergistically with GPX4 to provide enhanced protection against LPO and cellular injury. This synergy could underlie the observed anti-apoptotic and anti-inflammatory effects of 6M1@C60.

In conclusion, GPX4 is likely an important component of the pharmacological mechanism 6M1@C60, especially regarding its antioxidant and anti-ferroptotic effects. The interaction between 6M1@C60 and GPX4 may be a key factor underlying the compound’s protective actions across diverse biological contexts.

Based on the above information and your knowledge, please infer whether 6M1@C60 could influence ferroptosis in atherosclerosis by modulating GPX4. Provide only a single confidence score (0-100).

Response 1 (provided by GPT-4.0):

Based on the information provided and considering the critical role of GPX4 in ferroptosis and oxidative stress within atherosclerotic contexts, it is highly likely 6M1@C60 influences ferroptosis by modulating GPX4. Confidence score: 85.

### Evaluation of positive response rate

We defined a list of 27 key phrases indicative of negative or indeterminate responses to estimate the positive response rate of prompt-response pairs generated by LLMs. These phrases include: “am not able,” “am unable,” “cannot access,” “cannot conclude,” “cannot confirm,” “cannot find,” “cannot provide,” “cannot retrieve,” “cannot say,” “cannot state,” “cannot tell,” “couldn’t find,” “do not currently have,” “do not have,” “do not know,” “don’t have,” “don’t know,” “has not been determined,” “no documented evidence,” “no evidence,” “no known connection,” “no known direct association,” “no published research,” “no scientific evidence to,” “not able to provide,” “not clear,” and “not found to be directly involved.” A response was considered positive if none of the above phrases appeared in the generated answer.

### Western blot analysis

Whole-cell lysates were prepared using a universal radioimmunoprecipitation assay (RIPA) lysis buffer (Beyotime, Shanghai, China) supplemented with a protease inhibitor cocktail (Solarbio, China). After protein concentration was determined, equal amounts of protein were separated by 12% SDS-PAGE and transferred to 0.2 μm PVDF membranes (Merck Millipore, Ireland). Membranes were blocked with 5% non-fat milk for 1 h, followed by washing and incubation with primary antibodies overnight at 4 °C. After washing, membranes were incubated with HRP-conjugated secondary antibodies (Abcam, Cambridge, UK), diluted in TBST containing 5% non-fat milk, at room temperature. Protein bands were visualized using enhanced chemiluminescence (ECL) reagents (Thermo Fisher Scientific, USA). Band intensities were quantified using ImageJ software.

#### Cellular thermal shift assay (CETSA)

RAW264.7 cells were lysed using liquid nitrogen. After centrifugation, the supernatants were collected and incubated with 6M1@C60 (1, 2, 3, 4, or 5 µM) or solvent control (DMSO) at room temperature for 15 min. Samples were then aliquoted into 100 µL per tube and heated at a defined temperature range for 3 min, followed by cooling at room temperature for 3 min and placement on ice. After centrifugation, the supernatants were analyzed by Western blot.

### Statistical analysis

Statistical analyses were performed using GraphPad Prism 9 (GraphPad Software, USA). Comparisons among multiple groups were conducted by one-way analysis of variance (ANOVA) followed by Tukey’s post hoc correction. Data from three independent experiments are presented as mean ± standard error of the mean (SEM). A *p*-value less than 0.05 was considered statistically significant.

## Results

### Fullerenes and their derivatives may alleviate atherosclerosis by modulating ferroptosis

**Fig. 1:**
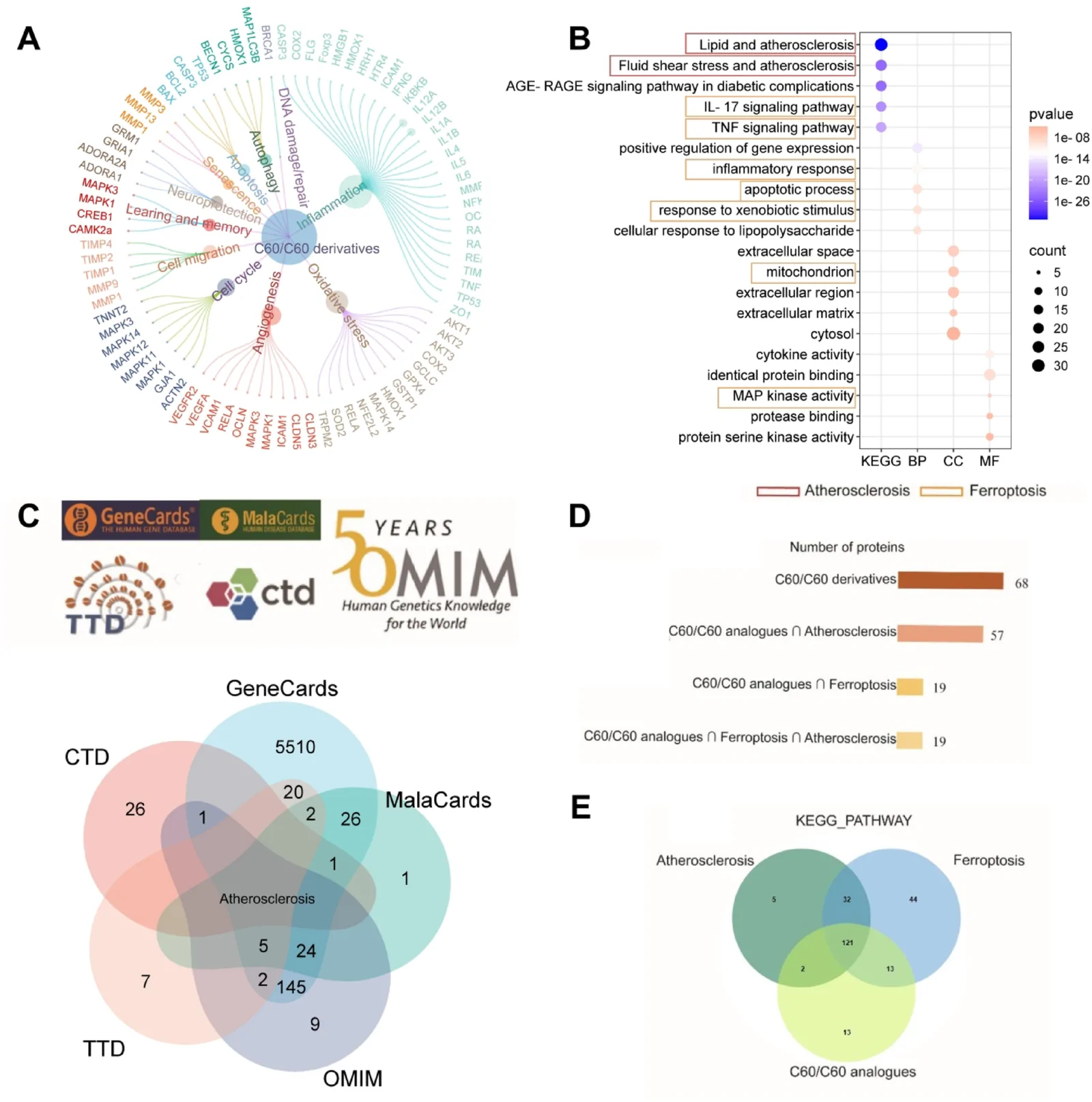
Potential modulation of ferroptosis by fullerenes and their derivatives in ameliorating atherosclerosis.Note: (**A**) Verified molecular targets of fullerenes and their derivatives; (**B**) Enrichment analysis of these targets; (**C**) Integration of atherosclerosis-related targets from GeneCards, MalaCards, OMIM, TTD, and CTD databases; (**D**) Correlation between the targets of fullerenes and atherosclerosis- and ferroptosis-related genes; (**E**) Pathway associations of fullerenes and their derivatives with atherosclerosis and ferroptosis

To identify the therapeutic targets of fullerenes, we collected experimentally validated protein targets associated with fullerenes and their derivatives. In total, 68 protein targets were compiled, which are involved in 11 biological processes, including inflammation, apoptosis, oxidative stress, autophagy, cell cycle, DNA damage/repair, angiogenesis, neuroprotection, aging, learning and memory, and cell migration [38–45] (Fig. 1A). For instance, Beyaz S et al. (2023) demonstrated that fullerene C60 mitigates acute lung injury induced by oxidative stress in rats via the cytochrome C/caspase-3/Beclin-1/IL-1α/HO-1/p53 signaling pathway [38]. Functional enrichment analysis of these targets suggested that fullerenes may attenuate the progression of atherosclerosis by modulating pathways related to inflammation, apoptosis, and oxidative stress and improving mitochondrial dysfunction. Ferroptosis, a programmed cell death dependent on iron, is characterized by mitochondrial morphological changes and metabolic dysregulation [46, 47]. These changes are closely linked to iron metabolism, including reduced mitochondrial membrane potential and decreased GSH levels [48]. Concurrently, oxidative stress and inflammation promote ferroptosis by generating ROS, LPO, and proinflammatory cytokines. These findings imply that fullerenes and their derivatives may influence ferroptosis-related mechanisms in atherosclerosis (Fig. 1B). To validate this hypothesis, we integrated atherosclerosis-related protein targets from GeneCards, TTD, MalaCards, OMIM, and CTD databases (Fig. 1C). We further substantiated our hypothesis by analyzing the overlap and correlations between fullerene-related targets and those associated with atherosclerosis and ferroptosis (Fig. 1D, E). Collectively, these results suggest that fullerenes and their derivatives may ameliorate atherosclerosis progression by regulating ferroptosis and improving inflammation and oxidative stress.

### 6M1@C60 Significantly Attenuates Atherosclerosis Progression and Suppresses Macrophage-Dependent Ferroptosis

In our previous study, we designed a hexagonal amphiphilic molecule (M1) with a hydrophobic hexaphenylbenzene core and six hydrophilic vinylpyridine groups [42]. A fullerene C60 molecule was encapsulated within the hydrophobic cavity of the M1 cage, thereby markedly enhancing the water solubility of C60. This complex was designated as 6M1@C60 (Figure S1). We successfully applied 6M1@C60 in a myocardial ischemia-reperfusion injury model, effectively reducing ROS levels, inhibiting cardiomyocyte apoptosis, and alleviating myocardial inflammation. Based on these findings, we hypothesized that 6M1@C60 may serve as a potential therapeutic candidate against atherosclerosis.

**Fig. 2:**
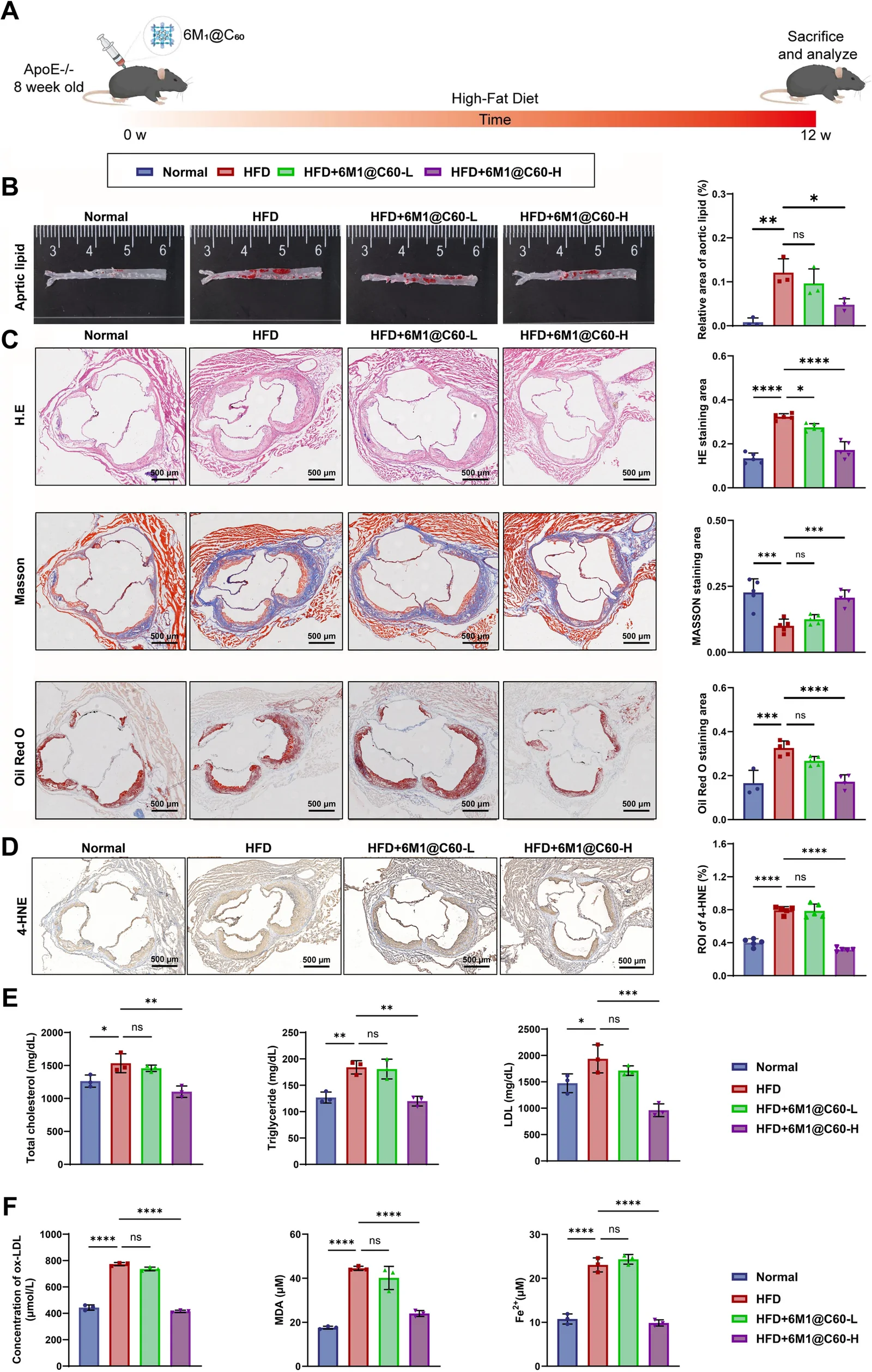
6M1@C60 significantly attenuates atherosclerosis progression and ferroptosis in ApoE^−/−^ mice.Note: (**A**) Schematic representation of the experimental design showing ApoE^⁻/⁻^ mice fed a HFD for anti-atherosclerosis investigation; (**B**) Representative Oil Red O-stained images of the aorta and quantification of atherosclerotic lesion area as a percentage of total aortic area; (**C**) Representative H&E, Masson’s trichrome and Oil Red O staining of the aortic root, with quantification of plaque burden, plaque collagen content and plaque lipid content as a percentage of total aortic area; scale ba *r* = 500 μm; (**D**) Quantification of 4-HNE-positive area by immunohistochemistry, expressed as a percentage of total area; scale bar = 100 μm; (**E**) Serum lipid levels including TC, TG, and LDL; (**F**) Quantification of serum ox-LDL, MDA, and Fe²⁺ levels across treatment groups. Data are presented as mean ± standard deviation (SD), *n* = 3–5. Statistical significance is indicated as: ****p < 0.0001, ***p < 0.001, **p < 0.01 vs. control group; *ns*: not significant

To evaluate the effect 6M1@C60 on atherosclerosis and ferroptosis, we employed an ApoE^−/−^ mouse model using 8-week-old male mice. The mice were randomly divided into four groups and subjected to different treatment regimens for 12 weeks: a Normal group, an HFD group, an HFD+6M1@C60-L (low-dose) group, and an HFD+6M1@C60-H (high-dose) group (Fig. 2A). En face, Oil Red O staining of the aorta revealed that high-dose 6M1@C60 markedly reduced the aortic plaque burden in ApoE^−/−^ mice (Fig. 2B). H&E, Masson’s trichrome, and Oil Red O staining of the aortic root showed that low-dose 6M1@C60 had limited effect on plaque formation, whereas high-dose 6M1@C60 significantly decreased the severity of atherosclerotic lesions and lipid accumulation within plaques (Fig. 2C). Furthermore, IHC analysis of 4-HNE, a marker of ferroptosis, indicated that high-dose 6M1@C60 substantially suppressed ferroptosis in the vascular system (Fig. 2D). Biochemical assays further confirmed that HFD feeding significantly increased TG, TC, LDL, ox-LDL, MDA, and Fe²⁺ serum levels. Administration of 6M1@C60 effectively reversed these HFD-induced changes in serum biomarkers (Fig. 2E-F).

**Fig. 3:**
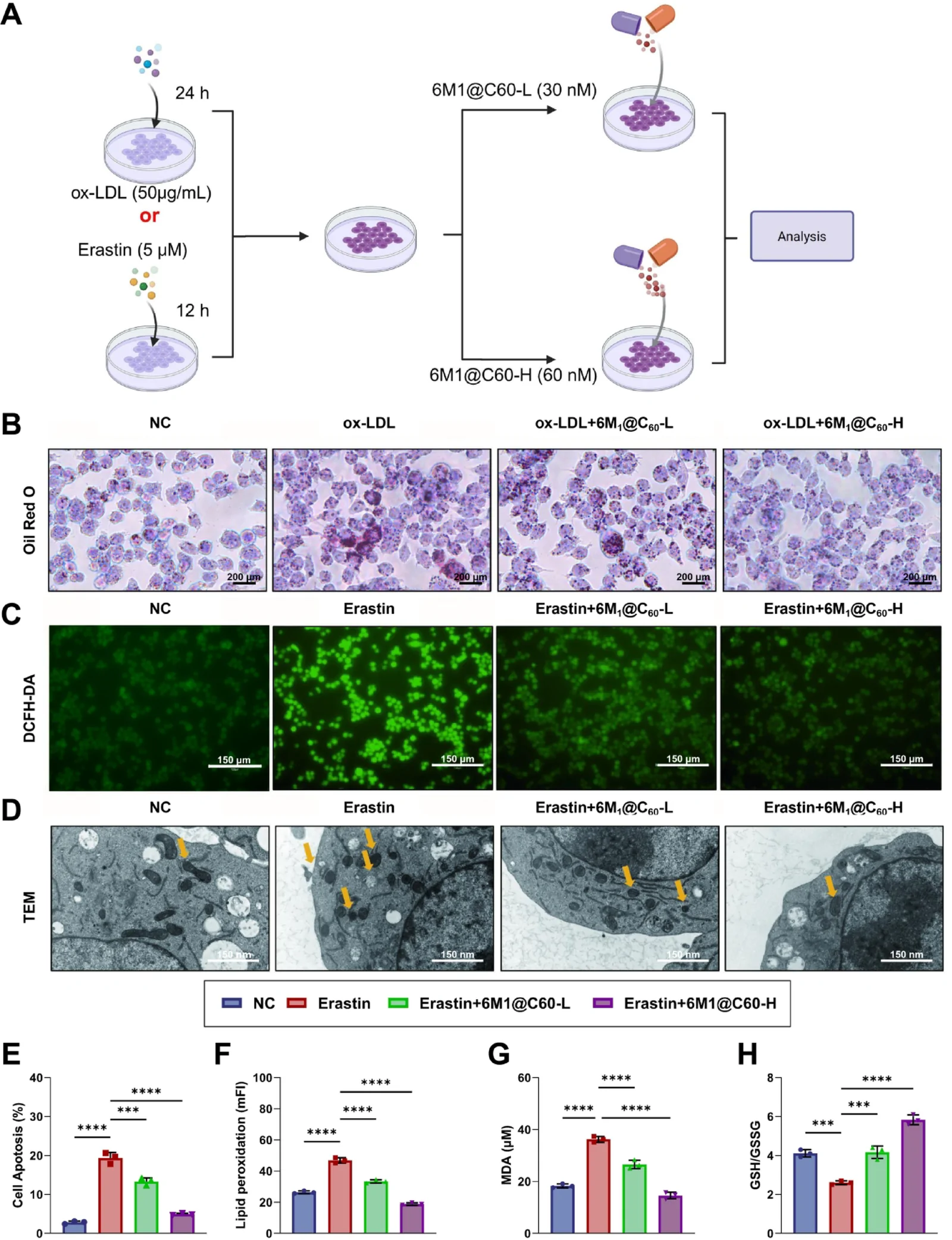
6M1@C60 Suppresses Macrophage Lipid Uptake and Macrophage-Dependent Ferroptosis.Note: (**A**) Schematic of the cell treatment protocol; (**B**) RAW264.7 macrophages were pretreated with ox-LDL (50 µg/mL) for 24 h, followed by treatment with low (30 nM) or high (60 nM) doses of 6M1@C60. Lipid accumulation was assessed by Oil Red O staining (scale bar = 200 μm); (**C**) After Erastin (5 µM) pretreatment for 12 h, RAW264.7 cells were incubated with 6M1@C60 (30 or 60 nM). Intracellular ROS levels were measured using the DCFH-DA fluorescent probe (scale bar = 150 μm); (**D**) TEM images of mitochondrial morphology in different treatment groups (scale bar = 150 nm); (**E**) Quantification of apoptotic cell populations; (**F**) Intracellular LPO levels; (**G**) MDA concentrations; (**H**) GSH/GSSG ratio. Data are presented as mean ± SD, *n* = 3–5. Statistical significance: ****p < 0.0001, ***p < 0.001 vs. control group

To further evaluate the therapeutic efficacy in vitro, RAW264.7 cells were pretreated with ox-LDL for 24 h, followed by the addition of low-dose (30 nM) and high-dose (60 nM) 6M1@C60 (Fig. 3A). Oil Red O staining revealed that macrophage lipid uptake was significantly reduced in the high-dose 6M1@C60 group compared with cells treated with ox-LDL alone (Fig. 3B). Furthermore, to assess the effects of 6M1@C60 on ferroptosis, RAW264.7 cells were pretreated with Erastin (an inducer of ferroptosis via inhibition of the cystine/glutamate antiporter System Xc-) for 12 h, followed by the addition of either low or high concentrations of 6M1@C60. DCFH-DA staining showed that 6M1@C60 reduced intracellular ROS levels dose-dependently (Fig. 3C). Ferroptosis is typically characterized by reduced mitochondrial volume and increased membrane density. TEM confirmed that high-dose 6M1@C60 reversed Erastin-induced mitochondrial shrinkage and membrane densification (Fig. 3D). To further validate the impact of 6M1@C60 on ferroptosis in RAW264.7 cells, we examined several ferroptosis-associated indicators, including cell death (PI staining), LPO, MDA levels, and the GSH/GSSG ratio. As expected, Erastin promoted cell death, LPO, and increased MDA levels while reducing the GSH/GSSG ratio in RAW264.7 cells. The addition of 6M1@C60 effectively reversed these effects (Fig. 3E-H).

During evaluation of the biological effects of 6M1@C60, immune-cell profiling was performed in blood, bone marrow and spleen. In the model group, CD8, CD68 and CD11b signals were significantly increased across these compartments, CD4 showed an increasing trend, and FOXP3 was significantly reduced; these changes were consistently reversed after 6M1@C60 administration, indicating restoration of immune tolerance, attenuation of pro-inflammatory responses, and suppression of myeloid-cell activation and infiltration during atherosclerosis progression (Figure S2A-C). Collectively, these data support an overall therapeutic benefit of 6M1@C60 in atherosclerosis and demonstrate its ability to effectively counteract erastin-induced, macrophage-dependent ferroptosis.

### Target Protein Prediction of 6M1@C60 Based on LLMs

Based on the analysis of experimentally validated targets of fullerene and its derivatives, 6M1@C60 is hypothesized to attenuate the progression of atherosclerosis by modulating ferroptosis, inflammation, and oxidative stress. We integrated experimentally validated targets of fullerene-related compounds (Figure S3A) with ferroptosis-related candidate targets associated with atherosclerosis (Figure S3B). Using ChatGPT-4.0 combined with CoT reasoning, we integrated protein function data and target information of fullerene derivatives to prioritize potential mechanisms of ferroptosis involvement in atherosclerosis and the association of candidate proteins with 6M1@C60. These predictions were used to hypothesize and score the likelihood of 6M1@C60 modulating ferroptosis in atherosclerosis via specific protein targets (Figure S3C). Ultimately, eleven high-confidence candidate targets were identified: IL-6, IL-1β, P53, RelA, HO-1, NRF2, GCLC, TXN, MAPK3, GPX4, and PARK7 (Figure S3D).

**Fig. 4:**
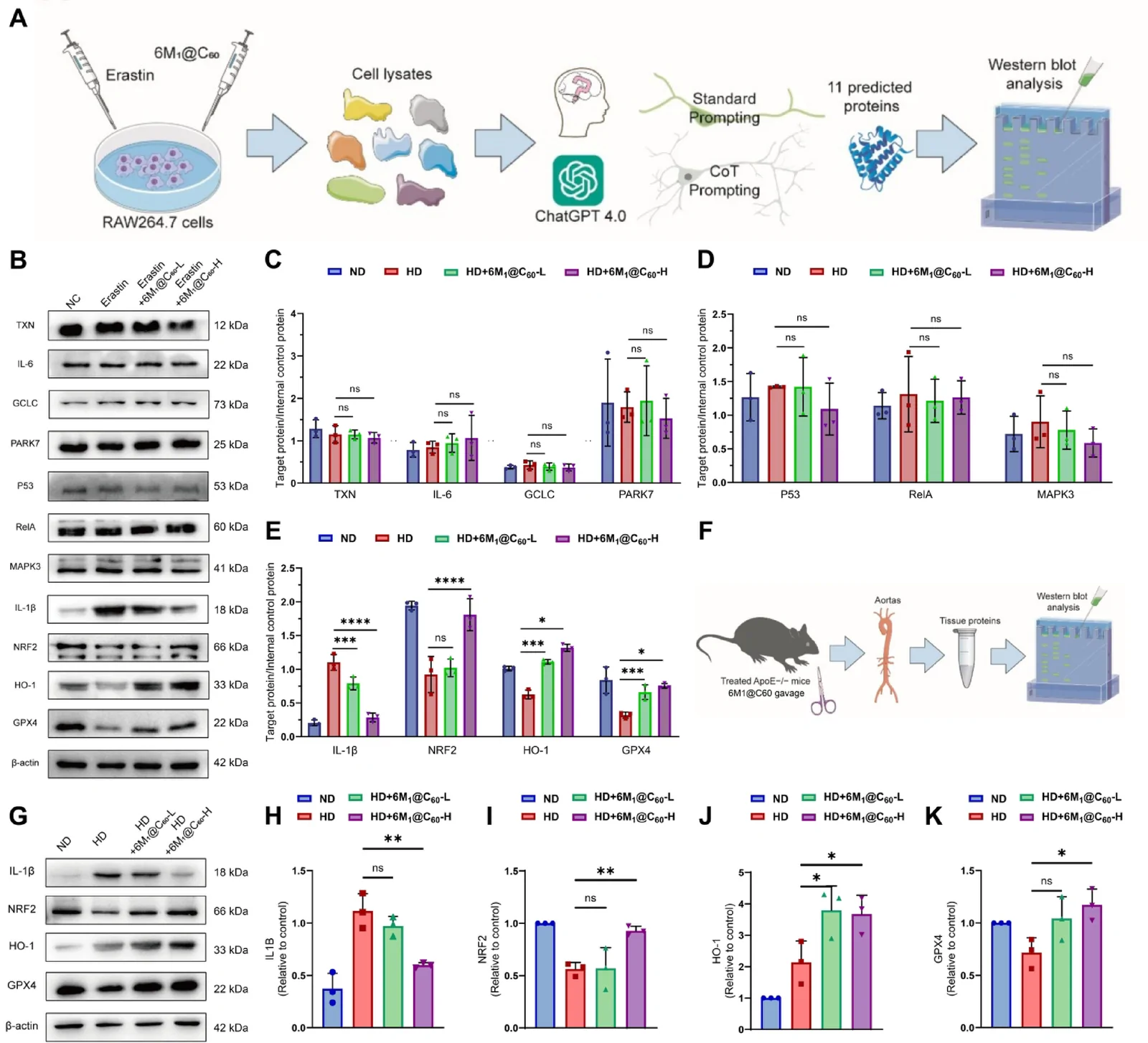
LLM-based prediction of 6M1@C60 target proteins.Note: (**A**) Schematic of the Western blot workflow for detecting expression of 11 proteins in RAW264.7 cells; (**B-E**) Representative immunoblot bands and quantitative analysis of TXN, IL-6, GCLC, PARK7, P53, RelA, MAPK3, IL-1β, NRF2, HO-1, and GPX4 expression in Erastin-pretreated cells; (**F**) Schematic of the workflow for western blot analysis of IL-1β, NRF2, HO-1 and GPX4 protein expression in mouse arterial tissues after oral gavage administration of 6M1@C60; (**G-K**) Representative blots and quantitative analysis of the above proteins in aortic tissue. Data are presented as mean ± SD, *n* = 3. Statistical significance: **p < 0.01, *p < 0.05 vs. control group; *ns* indicates no significant difference

We then validated the expression of these eleven proteins predicted by the LLM model using Western blot analysis (Fig. 4A-B). The results revealed that the expression levels of four proteins were significantly altered by 6M1@C60 treatment compared with the Erastin-induced model group (Fig. 4E), while the other seven proteins showed no significant changes (Fig. 4C-D). These four differentially expressed proteins (DEPs) were further validated in mouse aortic tissues using Western blot, and the in vivo results were consistent with the in vitro findings (Fig. 4F-K), suggesting the potential of LLMs to predict protein targets effectively. Among the DEPs, IL-1β was significantly downregulated, indicating that 6M1@C60 may exert its effects by suppressing ferroptosis-induced inflammatory signaling or cellular dysfunction. In contrast, NRF2, HO-1, and GPX4 were significantly upregulated. NRF2, as an upstream regulator of both HO-1 and GPX4, plays a pivotal role in regulating ferroptosis. Its elevated expression may initiate a cascade of downstream effects that influence redox homeostasis and cell ferroptotic activity.

### 6M1@C60 Modulates Erastin-Induced Ferroptosis via NRF2

**Fig. 5:**
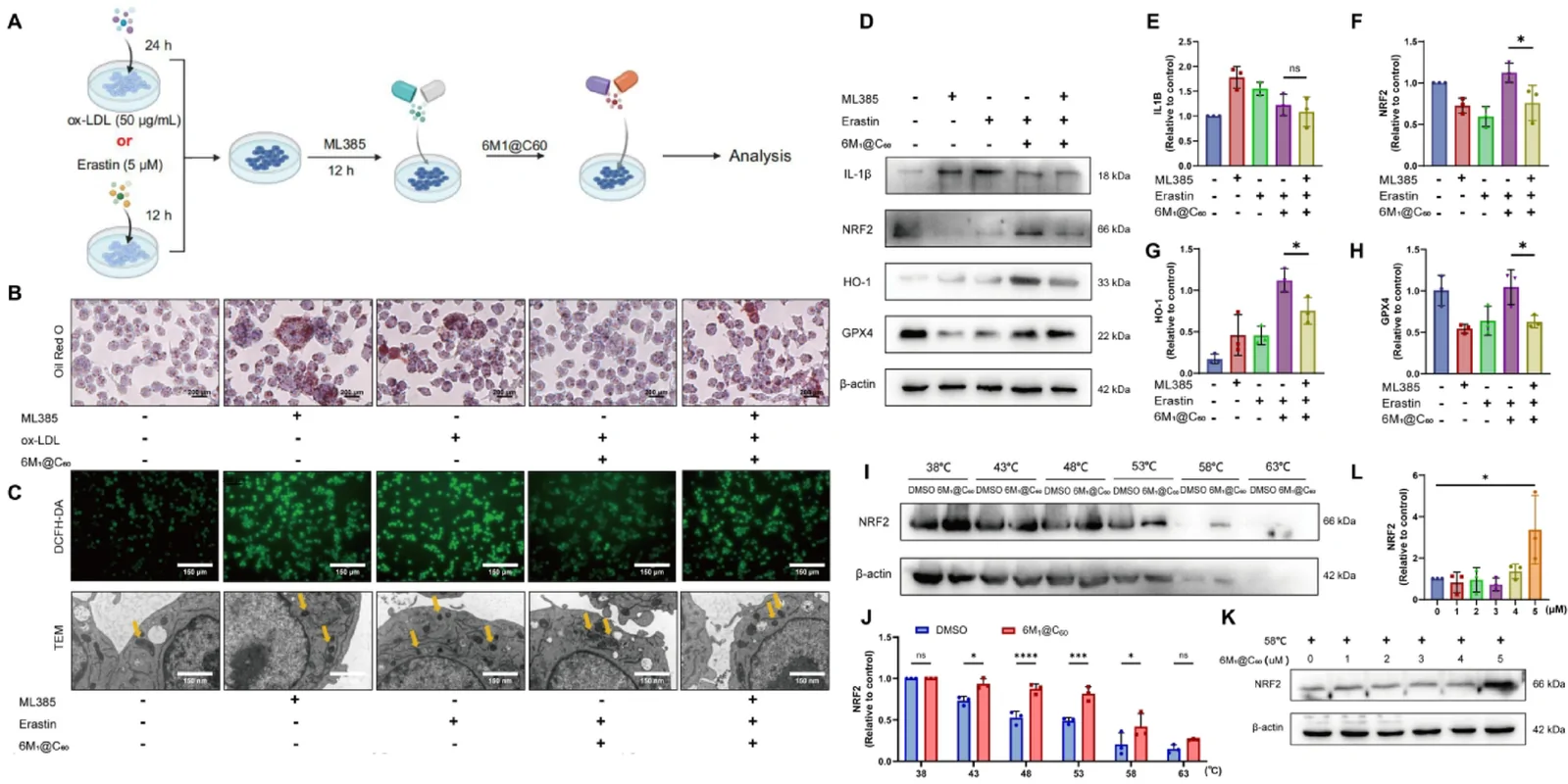
6M1@C60 regulates Erastin-induced ferroptosis via NRF2.Note: (**A**) Schematic of the cell treatment protocol; (**B**) Representative Oil Red O staining images of RAW264.7 cells pretreated with the NRF2 inhibitor ML385 (scale bar = 200 μm); (**C**) Representative ROS fluorescence images (scale bar = 150 μm) and mitochondrial morphology (scale bar = 150 μm) by TEM after ML385 pretreatment; (**D**) Representative immunoblot bands and quantitative analysis of IL-1β, NRF2, HO-1, and GPX4 expression in ML385-treated cells (**E-H**); (**I-J**) Immunoblot analysis showing the thermal stabilization of NRF2 by 6M1@C60 across different temperature gradients in RAW264.7 cells; (**K-L**) Effect of varying concentrations of 6M1@C60 on NRF2 stability at 58 °C. Data are presented as mean ± SD, *n* = 3. Statistical significance: ****p < 0.0001, ***p < 0.001, **p < 0.01, *p < 0.05 vs. control group; *ns* indicates no significant difference

To further investigate the role of NRF2 in lipid accumulation and ferroptosis, RAW264.7 macrophages pretreated with ox-LDL or Erastin for 12 h were incubated with the highly specific NRF2 inhibitor ML385 and subsequently treated with 6M1@C60 (60 nM) (Fig. 5A). Oil Red O staining revealed that ML385 significantly increased intracellular lipid accumulation and reversed the inhibitory effect 6M1@C60 on macrophage lipid phagocytosis (Fig. 5B). Consistently, DCFH-DA staining showed that ML385 attenuated the ROS-scavenging effect of 6M1@C60 against Erastin-induced oxidative stress. TEM further confirmed that ML385 treatment restored mitochondrial shrinkage and increased membrane density (Fig. 5C). Additional ferroptosis-related assays indicated that ML385 significantly reversed the protective effects of 6M1@C60 against Erastin-induced cell death, LPO, MDA accumulation, and GSH/GSSG imbalance (Figure S4).

Western blot analysis showed that while 6M1@C60 retained a strong suppressive effect on the inflammatory marker IL-1β following NRF2 inhibition, it no longer significantly upregulated HO-1 and GPX4 expression. These results suggest that NRF2 plays a pivotal role in mediating the ferroptosis-protective effects of 6M1@C60 (Fig. 5D-H). To determine whether 6M1@C60 directly binds to the NRF2 protein, we employed the CETSA based on the biophysical principle that ligand binding enhances protein thermal stability. To minimize the confounding effects of long-term incubation, which could lead to downstream metabolite interactions, the drug was incubated with cell lysates for only 1 min to assess direct binding. Western blot analysis showed that 6M1@C60 significantly increased NRF2 accumulation at temperatures ranging from 38 °C to 63 °C, indicating a direct interaction through thermal stabilization (Fig. 5I-J). To further validate the CETSA findings, lysates were treated at 58 °C with increasing concentrations of 6M1@C60. A dose-dependent enhancement of NRF2 thermal stability was observed (Fig. 5K-L).

To dissect the contribution of the NRF2–GPX4 axis to 6M1@C60 activity, NRF2 or GPX4 was individually silenced by siRNA. Upon NRF2 knockdown, 6M1@C60 still markedly reduced IL-1β, whereas induction of HO-1 and GPX4 was no longer evident (Figure S5A, B); accordingly, ARE reporter activity increased under erastin + 6M1@C60 but this increase was abolished in the presence of si-NRF2 (Figure S5C). Functionally, NRF2 deficiency exacerbated oxidative-stress phenotypes, including increased cell death, lipid peroxidation and MDA, together with a reduced GSH/GSSG ratio, and attenuated the protective effect of 6M1@C60 against erastin-induced injury (Figure S5D–G).

Under GPX4 knockdown, suppression of IL-1β and NRF2 by 6M1@C60 remained detectable, but its anti-ferroptotic protection was similarly weakened (Figure S5H–I, K–N). In the ARE reporter assay, si-GPX4 markedly altered the signal pattern under erastin + 6M1@C60 (Figure S5J), consistent with enhanced lipid peroxidation caused by loss of GPX4. In vascular smooth muscle cells, RSL3-induced ferroptosis showed a response to 6M1@C60 comparable to that observed in the erastin model (Figure S6A-N).

Together, these results support a model in which 6M1@C60 engages an NRF2-dependent transcriptional programme that regulates GPX4, thereby mitigating ferroptosis-associated phenotypes triggered by distinct inducers (erastin or RSL3).

### 6M1@C60 inhibits ferroptosis via the NRF2/HO-1/GPX4 axis

The NRF2/HO-1/GPX4 axis is a well-established regulatory pathway for ferroptosis. NRF2 activates downstream targets HO-1 and GPX4 to suppress LPO and ferroptosis. HO-1 catalyzes the degradation of heme into antioxidant byproducts such as carbon monoxide and biliverdin while simultaneously releasing Fe²⁺. GPX4 reduces lipid hydroperoxides using GSH, thereby maintaining membrane stability. Together, these molecules form an antioxidative barrier that prevents iron-dependent cell death. This regulatory axis has been extensively validated in cancer, cardiovascular diseases, neurodegenerative disorders, and metabolic syndromes studies. However, its role in atherosclerosis has rarely been reported.

**Fig. 6:**
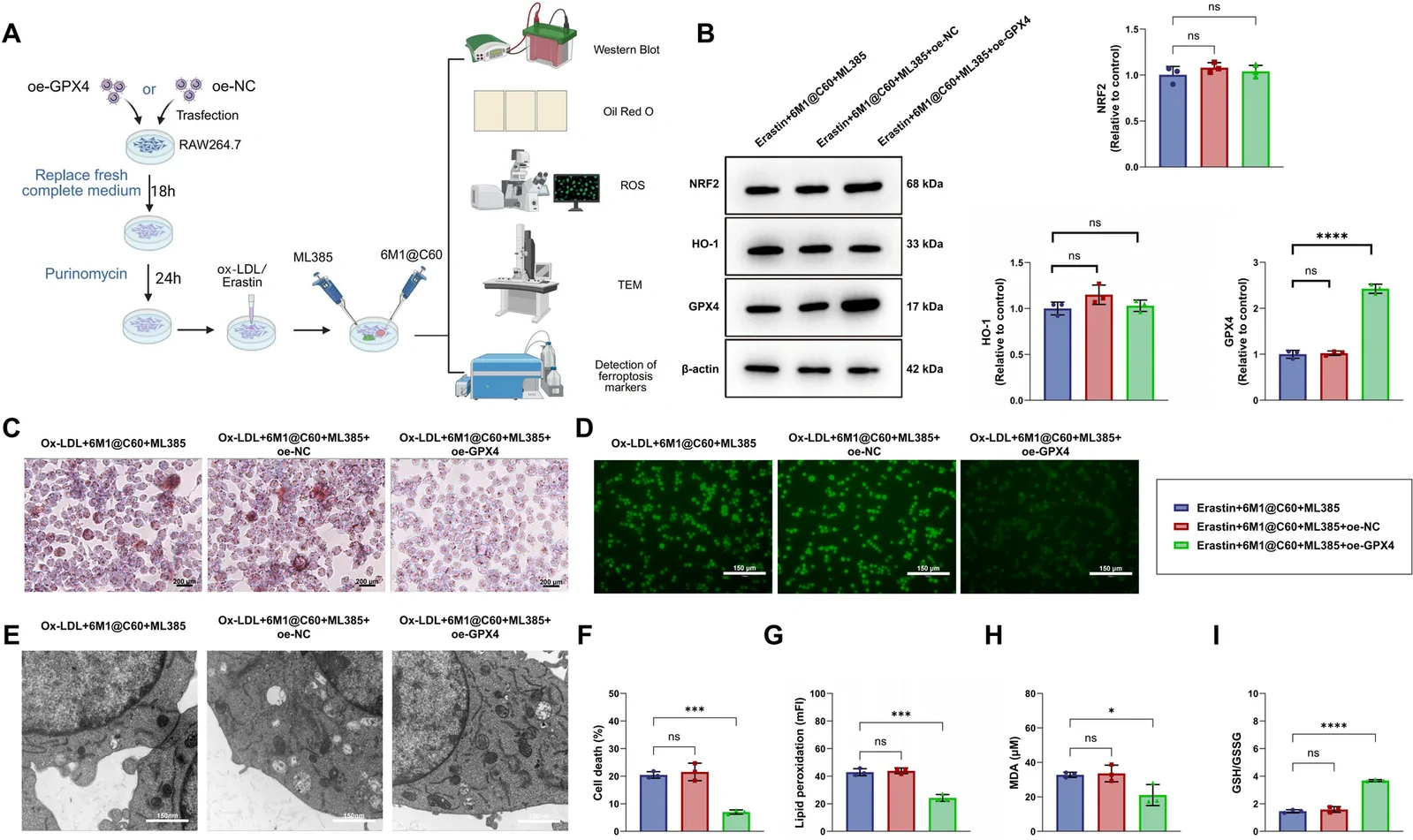
Effect of GPX4 overexpression on ferroptosis in RAW264.7 cells.Note: (**A**) Schematic diagram of the treatment protocol; (**B**) Western blot analysis of NRF2, HO-1, and GPX4 protein expression; (**C**) Oil Red O staining of intracellular lipid accumulation; (**D**) ROS levels measured using DCFH-DA fluorescence; (**E**) Mitochondrial morphology observed by TEM; (**F**) Apoptosis analysis; (**G**) Assessment of LPO levels; (**H**) Measurement of MDA levels; (**I**) Measurement of the GSH/GSSG ratio. Data are presented as mean ± SD, *n* = 3. Statistical significance: ****p < 0.0001, ***p < 0.001, *p < 0.05 vs. control group; *ns* indicates no significant difference

To investigate the regulatory relationship between 6M1@C60 and the NRF2/HO-1/GPX4 axis in ferroptosis, we infected RAW264.7 macrophages with lentivirus expressing oe-NC or oe-GPX4, followed by treatment with the NRF2 inhibitor ML385 after ox-LDL or Erastin pre-treatment for 12 h, in combination with 6M1@C60 administration (Fig. 6A). Western blot results showed that GPX4 overexpression significantly increased GPX4 protein levels in RAW264.7 cells, whereas NRF2 and HO-1 levels remained unchanged (Fig. 6B). Oil Red O staining demonstrated that lipid accumulation was significantly reduced in GPX4-overexpressing cells, indicating that GPX4 overexpression reversed the ML385-induced enhancement of lipid uptake in RAW264.7 cells (Fig. 6C). Similarly, DCFH-DA staining revealed that GPX4 overexpression counteracted the increase in ROS levels induced by ML385 (Fig. 6D). TEM showed that GPX4 overexpression restored mitochondrial morphology by increasing mitochondrial volume and reducing membrane density (Fig. 6E). In addition, ferroptosis-associated indicators demonstrated that GPX4 overexpression reversed the effects of ML385 on cell death, LPO, MDA levels, and the GSH/GSSG ratio (Fig. 6F-I).

In vascular smooth muscle cells, erastin treatment increased IL-1β and HO-1 protein levels, while reducing NRF2 and GPX4 protein levels, accompanied by decreases in NADPH content and SOD, CAT and GSH-PX levels; these changes were significantly reversed by 6M1@C60 administration (Figure S7A-B). These data indicate that 6M1@C60 alleviates erastin-induced oxidative stress and restores endogenous antioxidant capacity, thereby re-establishing redox homeostasis in vascular smooth muscle cells.

Our data reveal that 6M1@C60 suppresses ferroptosis through modulation of the NRF2/HO-1/GPX4 signaling axis.

### 6M1@C60 Alleviates Atherosclerosis in Mice via the NRF2/HO-1/GPX4 Axis

**Fig. 7:**
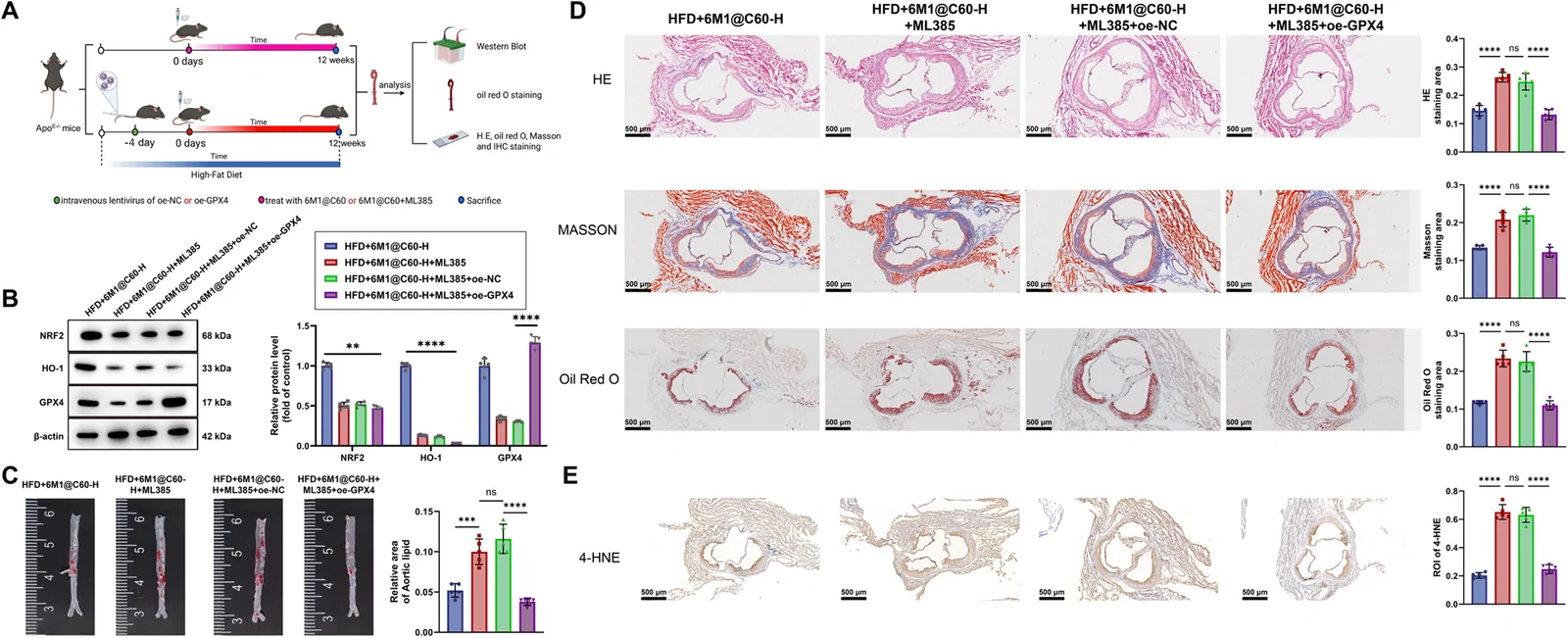
Effects of GPX4 overexpression and ML385 on the therapeutic efficacy 6M1@C60 in atherosclerotic mice.Note: (**A**) Schematic diagram of the treatment protocol; (**B**) Western blot analysis of NRF2, HO-1, and GPX4 expression in mouse aortic tissue; (**C**) Representative Oil Red O-stained images of the aorta and quantitative analysis of atherosclerotic lesions; (**D**) Representative H&E, Masson, and Oil Red O staining of the aortic root, with quantification of plaque burden, collagen content, and lipid content; (**E**) IHC staining of 4-HNE. Data are presented as mean ± SD, *n* = 5. Statistical significance: ****p < 0.0001, **p < 0.01 vs. control group; *ns* indicates no significant difference

To investigate the regulatory role of 6M1@C60 and the NRF2/HO-1/GPX4 axis in ferroptosis associated with atherosclerosis, we achieved GPX4 overexpression in vivo by tail vein injection of lentiviruses carrying oe-NC or oe-GPX4, followed by treatment with high-dose 6M1@C60 and the NRF2 inhibitor ML385 (Fig. 7A). Western blot analysis showed that ML385 treatment significantly reduced the protein levels of NRF2, HO-1, and GPX4 in mouse aortic tissues. Overexpression of GPX4 did not affect the expression of NRF2 or HO-1 but led to a significant increase in GPX4 protein levels (Fig. 7B). Oil red O staining of whole aortas revealed that ML385 markedly reversed the 6M1@C60-mediated reduction in aortic plaque burden in ApoE^−/−^ mice, whereas GPX4 overexpression again reduced the plaque burden (Fig. 7C). The aortic root’s H&E, Masson, and oil red O staining showed that ML385 significantly reversed the alleviating effect of 6M1@C60 on atherosclerosis severity in ApoE^−/−^ mice. GPX4 overexpression once again alleviated atherosclerosis and decreased lipid accumulation within the plaques (Fig. 7D). IHC results indicated that ML385 promoted 4-HNE protein expression in aortic tissue, while GPX4 overexpression reversed the ML385-induced increase in 4-HNE levels (Fig. 7E). In blood biomarker analysis, ML385 reversed the inhibitory effects 6M1@C60 on serum TG, TC, LDL, ox-LDL, MDA, and Fe²⁺ levels in ApoE^−/−^ mice. GPX4 overexpression once again reduced these serum indicators (Figure S8).

Consistently, we found that 6M1@C60 alleviates atherosclerosis in mice through modulation of the NRF2/HO-1/GPX4 axis.

### 6M1@C60 exhibits good biosafety both in vivo and in vitro

A key consideration for nanoparticle-based therapy is potential organ toxicity; therefore, systemic safety was evaluated at week 12 after treatment. Biodistribution analysis indicated a relatively uniform distribution of 6M1@C60 across major organs, with the highest signal detected in the heart (Table S3). Gross examination revealed normal organ appearance and size, and histological assessment of the heart, liver, spleen, lung and kidney showed preserved tissue architecture and normal cellular morphology, with no evident pathological alterations (Figure S9A). Consistently, serum AST, ALT, BUN and creatinine levels did not differ significantly among groups (Figure S9C), indicating no detectable impairment of hepatic or renal function. In vitro, exposure of RAW264.7 macrophages to increasing concentrations of 6M1@C60 did not affect cell viability (Figure S9B), further supporting a favourable safety profile.

## Discussion

This study aims to systematically investigate the mechanistic role of the functionalized fullerene derivative 6M1@C60 in atherosclerosis through AI-assisted target prediction and experimental validation, focusing on its potential regulation of ferroptosis. As a carbon-based nanomaterial, fullerene has shown promising therapeutic potential in various diseases due to its antioxidant and anti-inflammatory properties; however, its molecular targets and mechanisms of action remain poorly understood. Atherosclerosis, a chronic inflammatory disease closely associated with oxidative stress and programmed cell death, provides an ideal model for exploring the biomedical effects of fullerene. Previous studies have primarily focused on the material properties and in vitro antioxidant effects of fullerenes, lacking systematic mechanistic investigations. This study LLMs for target hypothesis generation and prioritization, and integrated in vivo and in vitro validation to establish a complete material-target-pathway framework, providing a new avenue for mechanistic dissection and translational development of fullerene-based therapeutics for cardiovascular disease.

Oxidative stress and ferroptosis are increasingly recognized as key drivers of atherosclerosis progression [49–51]. Iron accumulation, lipid peroxidation and elevated ROS can trigger endothelial dysfunction, inflammatory activation and plaque destabilization [25, 52, 53]. Ferroptosis is tightly shaped by cellular metabolic and redox states, and its susceptibility varies across cell types owing to inherent differences in iron handling, antioxidant defence networks and lipid peroxide detoxification capacity. Renal cell carcinoma–related models (e.g., HK-2) and highly mutated tumour cells (e.g., HT-1080) show heightened ferroptosis sensitivity linked to metabolic rewiring and increased oxidative burden [54]. In pathological settings, terminally differentiated cells such as neurons, cardiomyocytes and renal tubular epithelial cells represent prominent ferroptosis targets because of limited lipid peroxide clearance [55]. Macrophage ferroptosis is also state dependent; within atherosclerotic plaques, M1 macrophages with heightened inflammatory activity and lipid uptake are more prone to ferroptosis, thereby promoting necrotic core formation [56].

At the regulatory level, GPX4 is a central suppressor of ferroptosis, and loss of GPX4 activity is associated with aggravated arterial injury [27, 57, 58]. The NRF2 pathway is a major hub for antioxidant and cytoprotective responses and is considered protective in atherosclerosis [59, 60]. Prior work has largely focused on small molecules or natural products that modulate NRF2, whereas NRF2 has not been clearly established as a target in fullerene-based systems [61, 62]. Here, NRF2 emerged as a highly associated candidate target from database-based prediction and was supported by western blot and immunohistochemistry, strengthening its placement within the fullerene-mediated therapeutic mechanism. Mechanistically, 6M1@C60 may act through activation of NRF2 as an upstream regulatory node, thereby transcriptionally upregulating antioxidant and ferroptosis-related genes, including GPX4; this model is supported by reports showing that Nrf2 inhibition reverses resistance to GPX4 inhibitor–induced ferroptosis in head and neck cancer [63] and attenuates oestradiol-associated ferroptosis regulation and GPX4 upregulation.

Notably, 6M1@C60 exhibited significant anti-plaque and ferroptosis-inhibitory effects in this study, with consistent results observed in vivo (ApoE^−/−^ mice) and in vitro (RAW264.7 macrophages). Compared to previously reported studies employing unmodified fullerenes or other nanomaterials, 6M1@C60 represents a structurally optimized fullerene derivative with enhanced water solubility and biological stability, which may contribute to its superior antioxidant activity and target affinity in vivo. Earlier investigations into the use of fullerenes in atherosclerosis have largely focused on their anti-inflammatory properties or free radical scavenging capacity, with relatively little attention paid to cell fate mechanisms such as the regulation of ferroptosis [20]. This study strengthened mechanistic validation through molecular interventions, including NRF2 inhibition via ML385 and GPX4 overexpression, thereby establishing a clearer causal link between the nanomaterial and disease modulation.

The integration of AI is a major highlight of this study. Conventional target identification strategies often rely on differential gene expression analysis or protein-protein interaction networks, which are time-consuming, labor-intensive, and heavily dependent on investigator expertise [64, 65]. In contrast, LLMs, such as ChatGPT, offer capabilities in semantic understanding and logical reasoning, enabling the identification of potential targets by mining vast biomedical literature and databases and assisting in the construction of logical frameworks linking disease, material, and pathway [29, 30, 66]. This study significantly improved the efficiency and accuracy of target prediction by incorporating CoT reasoning and leveraging five authoritative databases (GeneCards, OMIM, TTD, MalaCards, and CTD). In line with recent advances in AI applications in drug discovery, such as AlphaFold for protein structure prediction, our findings demonstrate the translational potential of LLMs in mechanistic research on bioactive materials, contributing to developing AI-driven strategies for biomedical nanomaterial innovation.

From a materials science perspective, fullerene derivatives have recently shown multi-target regulatory potential in models of inflammatory bowel disease, diabetes, and neurodegenerative disorders [44, 67, 68]. Their π-conjugated structure endows them with excellent free-radical scavenging capacity, and the presence of multiple functional groups facilitates chemical modification and targeted regulation [69]. In this study, the derivative 6M1@C60 was specifically engineered to enhance hydrophilicity and biocompatibility, effectively overcoming the strong hydrophobicity and limited in vivo stability of conventional fullerenes. Given that ferroptosis and inflammation coexist in the microenvironment of atherosclerosis, the dual mechanistic actions of fullerenes align well with disease pathology. Therefore, fullerene materials may evolve beyond their classical antioxidant role to serve as multi-target intervention platforms, where AI-assisted prediction enables personalized and precise molecular design.

Unlike previous fullerene studies that mainly described functional outcomes without target localization or mechanistic proof, the present work establishes a clear material-target-pathway model. Specifically, 6M1@C60 activates the NRF2/HO-1/GPX4 signaling axis to modulate ferroptosis and oxidative stress, providing systematic molecular insight into fullerene action in cardiovascular disease. Earlier investigations inferred potential benefits primarily from reduced ROS levels; by contrast, the current study identifies the exact signaling pathway, offering stronger evidence and broader translational value. Moreover, we introduce an innovative research paradigm—AI-driven discovery of material mechanisms—which may promote deeper integration of AI and nanomedicine in future biomaterials research.

The results of this study suggest that fullerene-based nanomaterials may directly suppress ferroptosis within atherosclerotic lesions by neutralizing lipid peroxides, translating at the tissue level into reduced plaque formation. A plausible causal chain is that inhibition of ferroptosis preserves membrane integrity in lesional macrophages and vascular smooth muscle cells, thereby limiting the pro-inflammatory release of intracellular contents [70]. This cytoprotective effect could restrain plaque progression by curbing necrotic core expansion and dampening inflammatory amplification, and by maintaining foam-cell viability, which may favour plaque stabilization rather than a shift toward a vulnerable, necrotic phenotype [71].

Fullerene (C60) has been widely used as a nanoscale photosensitizer in tumour-associated photodynamic therapy [72]. However, clinical translation of fullerene-based therapeutics requires careful evaluation of key limitations, including long-term in vivo safety (for example, whether chronic, organ-specific accumulation leads to toxicity), biodistribution precision (whether sustained enrichment at atherosclerotic lesions can be achieved), and overall biocompatibility [20]. Although surface modification can improve aqueous dispersibility and reduce acute toxicity, potential long-term retention and immunogenicity of nanomaterials remain to be clarified through systematic preclinical toxicology studies.

This study, driven by AI-assisted target prediction and experimental validation, systematically explores the mechanism of the functionalized fullerene derivative 6M1@C60 in atherosclerosis. It proposes that 6M1@C60 may regulate ferroptosis by activating the NRF2/HO-1/GPX4 signaling axis, offering a novel perspective on material-mediated intervention in programmed cell death. Scientifically, this research enriches the mechanistic understanding of fullerenes in oxidative stress-related diseases. Technologically, it represents systematic application of LLMs in target prediction for biomaterials. Clinically, it provides theoretical support for the potential translational application of fullerene-based materials in cardiovascular disease. Several limitations should be acknowledged. First, the ApoE^−/−^ mouse model and RAW264.7 cells do not fully recapitulate human disease. Second, LLM-based predictions remain dependent on the quality and completeness of the underlying training data and may not capture the full complexity of biological systems, necessitating further experimental validation. Third, the long-term safety and potential adverse effects of 6M1@C60 have not been systematically assessed, and its upstream and downstream regulatory mechanisms require deeper investigation. Finally, although ML385 is a widely used Nrf2 inhibitor, potential off-target effects cannot be entirely excluded. Future work should extend to additional regulated cell-death pathways and establish a multi-material, multi-target, multi-technology framework for mechanism prediction and validation, incorporating genetic perturbation approaches such as siRNA- or CRISPR-Cas9-mediated knockdown to advance intelligent biomaterials for precision therapy.

## Conclusion

This study systematically elucidates the mechanism of action of the functionalized fullerene derivative 6M1@C60 in atherosclerosis by integrating LLMs with experimental validation. It demonstrates that 6M1@C60 significantly suppresses plaque formation and disease progression by activating the NRF2/HO-1/GPX4 signaling axis to modulate ferroptosis and oxidative stress. This work expands the mechanistic understanding of fullerene-based therapeutics in cardiovascular disease and demonstrates the practical utility of LLMs for hypothesis generation and mechanistic exploration in biomaterials research. The results provide a theoretical foundation for the rational design of fullerene-based materials and propose a novel paradigm of “AI-driven mechanistic discovery in materials research.” Although further investigation is warranted to evaluate its clinical safety and to expand mechanistic insights, this study lays a solid foundation for advancing intelligent biomaterials in precision therapeutics.

## Electronic Supplementary Material

Below is the link to the electronic supplementary material.

Supplementary Material 1.

## Data Availability

All data generated or analyzed during this study are included in this article and/or its supplementary material files. Further inquiries can be directed to the corresponding author.

## Abbreviations

AI: Artificial Intelligence
ApoE^−/−^: Apolipoprotein E Knockout
CCK-8: Cell Counting Kit-8
CETSA: Cellular Thermal Shift Assay
CoT: Chain-of-Thought
DAMP: Damage-Associated Molecular Pattern
DEP: Differentially Expressed Protein
DMSO: Dimethyl Sulfoxide
FBS: Fetal Bovine Serum
KEGG: Kyoto Encyclopedia of Genes and Genomes
GO: Gene Ontology
GPX4: Glutathione Peroxidase 4
GSH: Glutathione
GSSG: Glutathione Disulfide
H&E: Hematoxylin and Eosin
HFD: High-Fat Diet
HO-1: Heme Oxygenase 1
IHC: Immunohistochemistry
IOD: Integrated Optical Density
LDL: Low-Density Lipoprotein
LLM: Large Language Model
LPO: Lipid Peroxidation
MDA: Malondialdehyde
MOI: Multiplicity of Infection
NRF2: Nuclear Factor Erythroid 2-Related Factor 2
OCT: Optimal Cutting Temperature
ox-LDL: Oxidized Low-Density Lipoprotein
PBS: Phosphate-Buffered Saline
PI: Propidium Iodide
RIPA: Radioimmunoprecipitation Assay
ROS: Reactive Oxygen Species
SD: Standard Deviation
SEM: Standard Error of the Mean
TC: Total Cholesterol
TBA: Thiobarbituric Acid
TEM: Transmission Electron Microscopy
TG: Triglyceride
4-HNE: 4-Hydroxynonenal
