# Supplementary material for: LLMs uncover 6M1@C60’s therapeutic targets: inhibiting atherosclerotic progression via NRF2/HO-1/GPX4 axis-mediated ferroptosis suppression
Source: J Nanobiotechnology. 2026 Jun 2;24:793. doi: 10.1186/s12951-026-04474-3 (PMC13495479; doi:10.1186/s12951-026-04474-3)
Supplement: Supplementary file 1 [file 12951_2026_4474_MOESM1_ESM.docx]

**Figure S1. Schematic diagram of the structure and assembly of 6M1@C60.**

**Figure S2. 6M1@C60 modulates immune-cell composition and activation status.**

Note: Flow cytometry analysis of immune-cell profiles in (A) blood, (B) bone marrow and (C) spleen.

**Figure S3. Prediction of 6M1@C60 targets associated with ferroptosis in atherosclerosis based on prompt engineering.**

Note: (A) Experimentally validated targets of fullerenes and their derivatives; (B) Predicted candidate targets of 6M1@C60 by integrating targets related to atherosclerosis and fullerenes; (C) Evaluation of 6M1@C60-candidate targets-atherosclerosis relevance using standard prompts and CoT reasoning; (D) Identification of high-confidence protein targets

**Figure S4. Assessment of ferroptosis-related indicators.**

Note: (A) Apoptosis levels in each group; (B) LPO levels in each group; (C) MDA levels; (D) Changes in the GSH/GSSG ratio. Data are presented as mean ± SD, n = 3. Statistical significance: *****p* < 0.0001, ****p* < 0.001, **p* < 0.05 vs. control group; *ns* indicates no significant difference.

**Figure S5. 6M1@C60 modulates erastin-induced ferroptosis and redox homeostasis in vascular smooth muscle cells.**
Note: (A,B) Representative immunoblots (A) and quantification (B) of IL-1β, NRF2, HO-1 and GPX4 protein levels in cells pretreated with si-NRF2. (C) ARE luciferase reporter assay showing GPX4-associated reporter signal. (D) Apoptosis levels in each group. (E) Lipid peroxidation levels in each group. (F) MDA levels. (G) Changes in the GSH/GSSG ratio. (H,I) Representative immunoblots (H) and quantification (I) of IL-1β, NRF2, HO-1 and GPX4 protein levels in cells pretreated with si-GPX4. (J) ARE luciferase reporter assay showing GPX4-associated reporter signal. (K) Apoptosis levels in each group. (L) Lipid peroxidation levels in each group. (M) MDA levels. (N) Changes in the GSH/GSSG ratio. Data are presented as mean ± SD, n = 3. Statistical significance: *****p* < 0.0001, ****p* < 0.001, **p* < 0.05 vs. control group; *ns* indicates no significant difference.

**Figure S6. 6M1@C60 modulates RSL3-induced ferroptosis and redox homeostasis in vascular smooth muscle cells.**
Note: (A,B) Representative immunoblots (A) and quantification (B) of IL-1β, NRF2, HO-1 and GPX4 protein levels in cells pretreated with si-NRF2. (C) ARE luciferase reporter assay showing GPX4-associated reporter signal. (D) Apoptosis levels in each group. (E) Lipid peroxidation levels in each group. (F) MDA levels. (G) Changes in the GSH/GSSG ratio. (H,I) Representative immunoblots (H) and quantification (I) of IL-1β, NRF2, HO-1 and GPX4 protein levels in cells pretreated with si-GPX4. (J) ARE luciferase reporter assay showing GPX4-associated reporter signal. (K) Apoptosis levels in each group. (L) Lipid peroxidation levels in each group. (M) MDA levels. (N) Changes in the GSH/GSSG ratio. Data are presented as mean ± SD, n = 3. Statistical significance: *****p* < 0.0001, ****p* < 0.001, **p* < 0.05 vs. control group; *ns* indicates no significant difference.

**Figure S7. 6M1@C60 modulates ferroptosis-related proteins and redox homeostasis in vascular smooth muscle cells.**
Note: (A) Western blot analysis of IL-1β, NRF2, HO-1 and GPX4 protein levels. (B) ELISA-based quantification of NADPH, SOD, CAT and GSH-PX levels. Data are presented as mean ± SD, n = 3. Statistical significance: *****p* < 0.0001, ****p* < 0.001, **p* < 0.05 vs. control group; *ns* indicates no significant difference.

**Figure S8. Blood biochemical parameters in mice.**

Note: (A) Serum lipid levels (TC, TG, and LDL) in mice; (B) Levels of ox-LDL, MDA, and Fe²⁺ in each group after treatment. Data are presented as mean ± SD, n = 5. Statistical significance: *****p* < 0.0001 vs. control group; *ns* indicates no significant difference.

**Figure S9. Biosafety evaluation of 6M1@C60.**

Note: (A) H&E staining of the heart, liver, spleen, lungs, and kidneys (bar = 100 μm); (B) Cell viability following incubation with different doses of 6M1@C60. (C) Changes in serum AST, ALT, BUN and creatinine levels across groups. Data are presented as mean ± SD, n = 3. Statistical significance: *ns* indicates no significant difference compared with the control group.

**Table S1. 6M1@C60 safety evaluation in the mice**

|  | Control | 6M1@C60(20mg/L) | 6M1@C60(40mg/L) |
| --- | --- | --- | --- |
| ALT(U/L) | 28.68±2.56 | 31.28±2.23 | 32.47±2.12 |
| AST(U/L) | 42.17±3.33 | 46.45±1.29 | 45.37±0.67 |
| BUN(mM/L) | 1.20±0.16 | 1.41±0.04 | 1.55±0.13 |
| CRE(mM/L) | 101.10±7.51 | 107.50±4.35 | 99.39±6.28 |

**Table S2 si-RNA sequences**

| **Gene** | **Sense (5′→3′)** | **Antisense (5′→3′)** |
| --- | --- | --- |
| Si-GPX4 | GGAUGAAAGUCCAGCCCAATT | UUGGGCUGGACUUUCAUCCTT |
| Si-NRF2 | AGACAUAGAUCUUGGAGUATT | UACUCCAAGAUCUAUGUCUTT |
| Si-NC | UUCUCCGAACGUGUCACGUTT | ACGUGACACGUUCGGAGAATT |

**Table S3. The biological distribution of 6M1@C60 in the major organs of mice.**

| **No.** | organs | Distribution(%) |
| --- | --- | --- |
| 1 | Heart | 29.03 |
| 2 | Liver | 16.39 |
| 3 | Spleen | 15.58 |
| 4 | Lung | 17.24 |
| 5 | Kidney | 21.76 |
